# Physicochemical Properties of Biochar Produced from Goldenrod Plants

**DOI:** 10.3390/ma15072615

**Published:** 2022-04-02

**Authors:** Bogusława Łapczyńska-Kordon, Zbigniew Ślipek, Karolina Słomka-Polonis, Jakub Styks, Tomasz Hebda, Sławomir Francik

**Affiliations:** 1 Department of Mechanical Engineering and Agrophysics, Faculty of Production Engineering and Energetics, University of Agriculture in Krakow, Balicka 120, 30-149 Krakow, Poland; karolinaslomka@gmail.com (K.S.-P.); jakub.styks@urk.edu.pl (J.S.); tomasz.hebda@urk.edu.pl (T.H.); slawomir.francik@urk.edu.pl (S.F.); 2 Technical Institute, State Higher Vocational School, Staszica 1, 33-300 Nowy Sącz, Poland; zbigniew.slipek@urk.edu.pl

**Keywords:** goldenrod, biochar, biomass, torrefaction, physicochemical properties

## Abstract

Torrefaction is one of the methods of thermal treatment of biomass, which allows obtaining a product of better quality in the form of biochar. The aim of the paper was to analyze the possibility of using goldenrod *(Solidago canadensis*, *Solidago gigantea*) for the production of biochar. The torrefaction process involved the vegetative and generative parts as well as the whole plant at temperatures of 250 °C and 275 °C, for 3 h. Next, the physicochemical properties of the raw material and biochar were determined, namely moisture content, ash content, volatile matter content, calorific value, and heat of combustion. The bulk density of raw biomass and biochar was also determined. It was found that after biomass torrefaction, the ash content, calorific value, and heat of combustion increased, while volatile matter content decreased. It has been observed that in both the case of raw biomass and biochar, the plant species and the sampled parts have a significant impact on the ash content, volatile matter content, calorific value, and heat of combustion.

## 1. Introduction

In recent years, during the search for energy sources that would have a less negative impact on the environment (especially CO_2_ emissions), biomass has emerged as a real alternative to solid fossil fuels (e.g., coal and lignite) [1,2]. 

Biomass has great technical and development potential and is one of the main sources of renewable energy, ranking third in size and availability [3]. However, its physicochemical properties make biomass problematic during combustion, mainly due to its moisture, bulk density, and flash point. This is of great importance, especially when co-firing with coal. Raw biomass is characterized by high moisture content, which results in it having a low calorific value [3,4].

To improve the energy use of biomass, it is subjected to various processing and treatments [1,5,6]. One of the main methods of biomass processing is thermal treatment. Depending on the temperature used and the operating conditions, thermal processes can be divided into drying, torrefaction, pyrolysis, and gasification. Thermal treatment of biomass can improve energetic, physico-mechanical, and chemical properties [7,8].

Torrefaction is one of the methods of thermolysis, which is defined as the chemical decomposition of substances caused by elevated temperature [6,9,10]. The torrefaction process involves the slow roasting of biomass at a temperature of 220–350 °C without air access, at a pressure close to atmospheric [6,8,11,12]. If the preset temperature range of the process is maintained, there is a greater loss of oxygen and hydrogen as compared to the loss of carbon. Lowering the oxygen to carbon ratio improves the properties of the biochar obtained as a result of this process [6,13,14]. It was also found that in the process of torrefaction of plant biomass, energy density increases and mass decreases [15,16,17]. Exceeding either the temperature of 350 °C during roasting or extending the processing time is inadvisable as this leads to the formation of charcoal. Biochar comminution is also better, which is important for combustion in fluidized bed furnaces [11]. Biochar is a homogeneous material, characterized by hydrophobic properties and increased resistance to biological agents compared to unprocessed biomass [2].

Biochar is used not only as a new generation fuel in the energy sector [7], due to its particular properties, it is also used in agriculture (the water retention capacity of soil, greenhouse production, composting of organic substances, reduction of unpleasant odors), construction (as an insulation material, ecological construction), environmental protection (reclamation of sewage sludge carbonization landfill, removal of pollutants from aqueous solutions and gases, reclamation of soil contaminated with organic and inorganic substances) [7,18]. These are only some of the most important directions for the use of biochar; however, the possibilities of using this material are much wider [7,14,19]. Many other advantages of using biochar are described in the literature relating to the use of renewable energy sources for the easy recovery of metals [20] and its use in fuel cells [21].

Biochar, as an ecological, porous carbon material produced from raw biomass by pyrolysis, has recently also been proposed as support for powdered photocatalytic platforms [19,22,23]. The main advantage of using biochar is that it allows for a significant reduction in the environmental footprint and the costs associated with the synthesis of the photocatalyst compared to traditional materials based on noble metals and carbon. Its production is also highly sustainable because the waste biomass can be used as a raw material [24,25].

Biochar obtained as a result of the torrefaction process may significantly differ in its properties, which depend on the type and origin of the biomass, the temperature of the process, its duration, and the initial moisture content. It has been observed that increasing the torrefaction temperature and extending the duration of the process increases the calorific value of the biochar. The higher the moisture content in the torrefied biomass, the greater the energy loss in the product [15,16,26].

Various types of plant and waste biomass from agro-food processing and municipal waste were subjected to the torrefaction process. Such a potential raw material for the production of biochar may be the family of *Asteraceae* plants, including goldenrod. 

Nguyen et al. [27] presented the results of research on the thermal processing of *Asteraceae* plants (including goldenrod) into biochar, and their properties depending on the process parameters. The goldenrod pyrolysis process was carried out at a temperature of 450 °C in a nitrogen atmosphere, with the obtained product to be used in soil [27]. The influence of the processing temperature on the physicochemical properties of biochar which would be suitable for use in cadmium adsorption was also investigated [28].

Goldenrod belongs to the *Asteraceae* family, comes from North America, and is the most common invasive plant in Europe [29,30]. Goldenrods colonize wastelands and arable green areas, especially where the use of turf was abandoned by grazing or mowing. Goldenrod is characterized by high plasticity and adapts to various habitat conditions by increasing the growth of shoots and inflorescences as well as strengthening the rhizomes. It is also resistant to extreme habitat conditions and distinguished by its rapid growth and high invasiveness [29,31]. It seems, therefore, that due to the high fertility of this plant its cultivation for energy purposes is justified. The more so as the use of biomass of various origins may, in Polish conditions, make a significant contribution to the achievement of the assumed goals of reducing carbon dioxide emissions and producing energy and heat from renewable fuels [1,2].

Hence, the study aims to assess the physicochemical properties of biochar produced from the whole plant and the vegetative and generative parts of the plant from two goldenrod species (*Solidago canadensis* and *Solidago gigantea*).

Determining these properties will enable evaluating the feasibility of using goldenrod in the production of biochar, having applications in agriculture, environmental protection, and as a potential eco-fuel in energy.

## 2. Materials and Methods

### 2.1. Characteristics of the Biomass Material

The research covered the *Giant* Goldenrod cultivar (*Solidago gigantea*) and the *Canadian* cultivar (*Solidago canadensis*). Plants were divided into vegetative and generative parts, and each of the listed parts as well as the whole plants were subjected to laboratory tests before and after the torrefaction process. To compare the input material with the produced biochar, physicochemical and technical analyses were conducted whereby moisture content, bulk density, ash content, volatile matter content, combustion heat, and calorific value of the tested samples were determined.

### 2.2. The Torrefaction Process

The torrefaction process was carried out in a muffle furnace (type Snol 8.2/1100) at temperatures of 250 °C and 275 °C, respectively. First, the tested biomass was ground. Then, the mass of the crucible and mini-generator before and after filling with the tested material was determined. The filled mini-generator, equipped with a hole for discharging gaseous products formed during roasting, was placed in a muffle furnace and heated to the assumed temperature. The sample was subjected to thermal conversion—roasting in an oven for 3 hours. After completion of the process, the product crucible was cooled to ambient temperature and reweighed on an analytical balance.

### 2.3. Measurement of Moisture Content

Material moisture content was determined in accordance with EN ISO 18134-3:2015-11 by the indirect drying method [32]. A 1 g sample of the material to be tested was dried in a laboratory oven with forced air circulation and thermostatic temperature control, at a temperature of 110 °C for 90 min. After drying was completed, the samples were placed in a desiccator and, after cooling, weighed on an analytical balance (accuracy ± 0.0001 g). Moisture measurements were made on the ground samples below 0.2 mm. The moisture content (*MC*) was calculated from the following relationship:

(1) $$MC=\frac{m_{p}-m_{ss}}{m_{p}}\cdot 100\%$$

where: *m_p_*—initial sample mass, kg; *m_ss_*—dry substance mass, kg.

### 2.4. Measurement of Bulk Density

The bulk density was determined based on the relationship:

(2) $$\rho_{f}=\frac{m_{1}-m_{0}}{V}$$

where: *m*_0_—mass of crucible, kg; *m*_1_—mass of crucible with sample, kg; *V*—measuring cylinder volume, m^3^.

For this purpose, the mass of a cylinder with a volume of 100 mL and the mass of the cylinder filled with the comminuted test material with a particle size of 0.2 mm were determined. Based on the obtained measurements, the bulk density was calculated from Equation (2).

### 2.5. Measurement of Ash Content

The determination of the ash content in the tested material was made in accordance with the EN ISO 18122:2016-01 standard [33], using a muffle furnace SNOL 8.2/1100. The Ash content was determined by a slow incineration process, by gradually heating (60–80 min) to a temperature of 550 °C, and then calcining a sample of 1 g in the crucible to a constant mass at this temperature. After removing the crucibles, they were cooled in the desiccator to room temperature and weighed using an analytical balance (±0.0001 g).

### 2.6. Measurement of Volatile Matter Content

Volatile matter content was determined in accordance with EN ISO 18123:2016-0 [34]. The measurement was made by roasting a sample of the tested material weighing 1 g in closed porcelain crucibles at 850 °C for 7 min, without air access. After the process was completed, the crucibles were placed in the desiccator, cooled to ambient temperature, and then weighed on an analytical balance (±0.0001 g).

### 2.7. Combustion Heat and Calorific Value Measurement

The heat of combustion and the calorific value was determined by the bomb calorimeter in accordance with EN ISO 18125:2017-07 using the KL-12 calorimeter [35]. Samples of 1 g, weighed using an analytical balance (±0.0001 g), in the form of a pellet with embedded cantal wire were placed in a bomb calorimeter with poured distilled water (0.002 dm^3^), then the bomb was filled with oxygen and sealed. After placing the bomb in a calorimetric crucible filled with water, the ignition system was turned on and temperature changes in the system were recorded on the computer. The computer program automatically calculated the heat of combustion *Q_s_* based on the heat balance of the calorimeter and the calorific value *Q_w_* from the equation:

(3) $$Q_{w}=Q_{s}-H_{p}\cdot \left(9\cdot h^{r}+w\right)$$

where: *H_p_*—the heat of water evaporation, J∙kg_H2O_^−1^, *h^r^*—a mass fraction of hydrogen in the working condition of biofuel, kg∙kg^−1^, *w*—a mass share of total moisture in biofuel, kg∙kg^−1^.

### 2.8. Statistical Analysis

To check whether the analyzed factors have an impact on the observed variables (testing statistical hypotheses), the Analysis of Variance (ANOVA) was used [36,37].

ANOVA is one of the most popular methods of statistical inference in the case of comparing more than two groups.

Tang et al. [28] used one-way ANOVA (one-way analysis of variance) in the tested biochar derived from *Solidago canadensis* produced at temperatures of 400–600 °C.

ANOVA was also used for the statistical analysis of thermochemical processes with biochar derived from plants of the *Asteraceae* family, which also includes goldenrod. For example, Silva et al. [38] used ANOVA for statistical analysis in biomass studies of *Flourensia oolepis* in terms of the influence of temperature on product yields in various plant organs (leaves and stems). Wang et al. [39] used one-way and two-way analyses of variance in studies of biochars obtained from invasive plants at different temperatures (300 °C, 500 °C, and 700 °C) (biochars derived from five invasive plants at different temperatures). Two of these plants (*Bidens pilosa L*. and *Mikania micrantha*) belong to the *Asteraceae* family.

ANOVA was also used in research on the production of biochar, bio-oil, and biogas from sugarcane bagasse [40], torrefaction of palm kernel shells [41,42], torrefaction at temperatures from 200 °C to 300 °C of three types of *Jatrofy* biomass [43], and wet torrefaction of Korean pine [27].

Two independent ANOVAs were carried out for biomass and biochar from goldenrod. These analyses were performed for four dependent variables: ash content [%], volatile matter content [%], calorific value [MJ∙kg^−1^], and heat of combustion [MJ∙kg^−1^].

A two-way ANOVA was performed for the biomass. The intergroup factors (independent variables) were species (two levels: *Giant* goldenrod, *Canadian* goldenrod), and plant parts (three levels: whole plant, vegetative part, generative part).

A three-way ANOVA was performed for biochar. The intergroup factors (independent variables) were species (two levels: *Giant* goldenrod, *Canadian* goldenrod), plant parts (three levels: whole plant, vegetative part, generative part), and material (two levels: 275 °C and 250 °C).

Before this, it was determined whether the main assumptions concerning normal distribution of dependent variables within groups as well as homogeneity of variance were met [44]. Normal distribution was verified by the Shapiro-Wilk test while homogeneity of variance was verified by Levene’s test.

In case the null hypothesis is rejected based on the ANOVA results (no significant differences between the groups), post-hoc tests (multiple comparison tests) must be performed. These tests allow classifying the means to significantly different groups [36]. To determine homogeneous groups, the Fisher’s Least Significant Difference (LSD) test was used.

Statistical analysis was carried out using the Statistica software (Dell Inc (Tulsa, OK, USA, 2016). Dell Statistica (data analysis software system), version 13. software.dell.com). Results were expressed as the means ± Standard Deviations (SD). Values of *p* < 0.05 were considered statistically significant.

## 3. Results

The analysis concerned the influence of the aforementioned plant species and its parts (vegetative or generative) on the ash content and volatile matter content as well as the calorific value and heat of combustion. In the case of biochar, the analysis also concerned the influence of the temperature at which the torrefaction process was carried out.

### 3.1. Biomass and Biochar Bulk Density

The bulk density of the biomass of *Giant* goldenrod and *Canadian* goldenrod ranges from 331 to 487 kg∙m^−3^. The bulk density of the produced biochar ranged from 149 to 324 kg∙m^−3^. The individual values depending on the species as well as the plant parts and plant material are presented in Table 1.

### 3.2. Moisture Content in Biomass and Biochar

The moisture content of the tested biomass ranged from 9.18% to 12.86% and depended on the species and plant parts. For *Giant* goldenrod—whole plant the moisture content ranged from 9.18% to 10.73% and was the lowest among the tested species and plant parts. The other tested samples were characterized by a similar range of changes in moisture content (from approx. 11% to approx. 13%) (Table 2).

The moisture content of biochar obtained at 250 °C ranged from 5.28% to 8.96%. The lowest moisture content was found in biochar obtained from the *Giant* goldenrod—whole plant, and the highest in the *Giant* goldenrod—generative part (Table 2). The moisture content obtained at 275 °C ranged from 5.19% to 9.37%. The lowest moisture content was again obtained from the *Giant* goldenrod, whole plant, and the highest was again found in the *Giant* goldenrod, generative part. It was observed that the level of moisture determined for biomass and biochar obtained during roasting at two different temperatures for the whole plants and their generative and vegetative parts was similar for both species (*Giant* goldenrod and *Canadian* goldenrod)—the lowest for the whole plant, and the highest for the generative part of both species.

### 3.3. Statistical Analysis Results

For all dependent variables, both for biomass and biochar, the test results for individual groups showed the assumptions concerning the normal distribution (Shapiro-Wilk test) and homogeneity of variance (Levene’s test) at the significance level of *p* = 0.05 are met.

**Figure 1:**
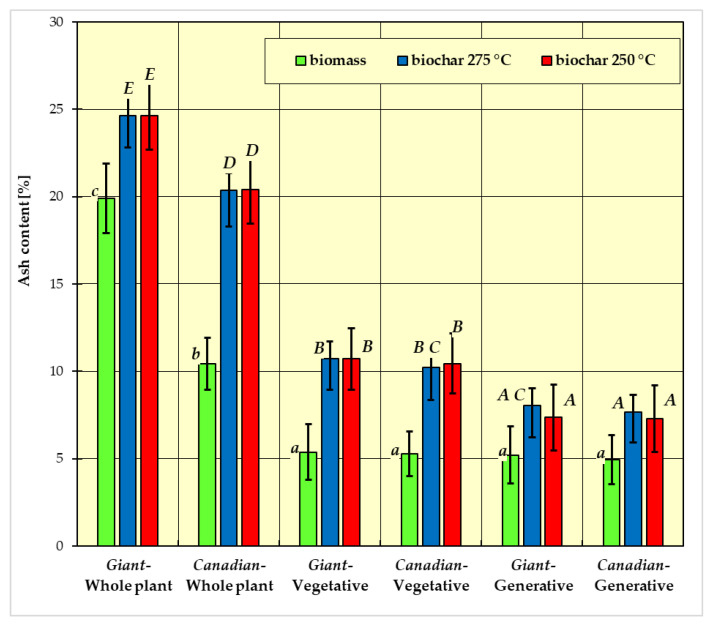
Average ash content in biomass and biochar. All data are expressed as mean ± SD. Bars with a different letter indicate significant differences according to Fisher’s LSD test (*p* < 0.05 was accepted as statistically significant). Homogeneous groups are marked with the same letters (lowercase letters *a*, *b*, *c* for biomass homogeneous groups, uppercase letters *A*, *B*, *C*, *D*, *E* for biochar homogeneous groups).

**Figure 2:**
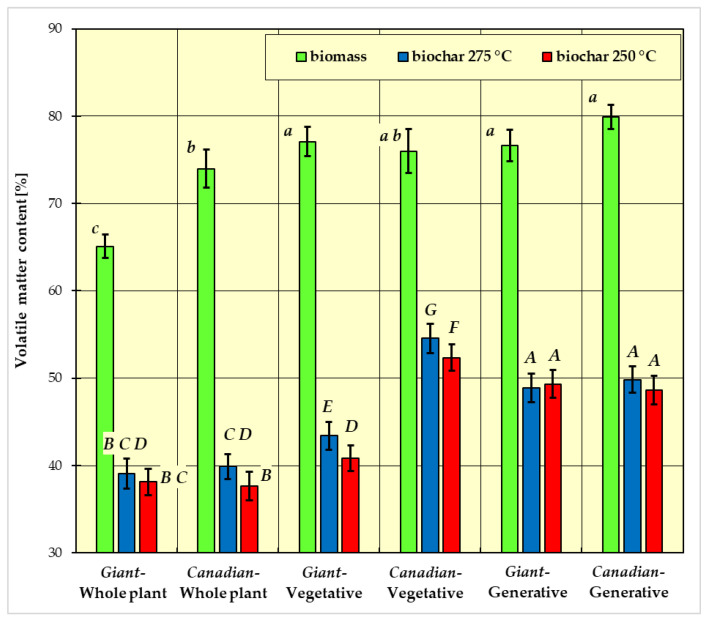
Average volatile matter content in biomass and biochar. All data are expressed as mean ± SD. Bars with a different letter indicate significant differences according to Fisher’s LSD test (*p* < 0.05 was accepted as statistically significant). Homogeneous groups are marked with the same letters (lowercase letters *a*, *b*, *c* for biomass homogeneous groups, uppercase letters *A*, *B*, *C*, *D*, *E*, *F*, *G* for biochar homogeneous groups).

**Figure 3:**
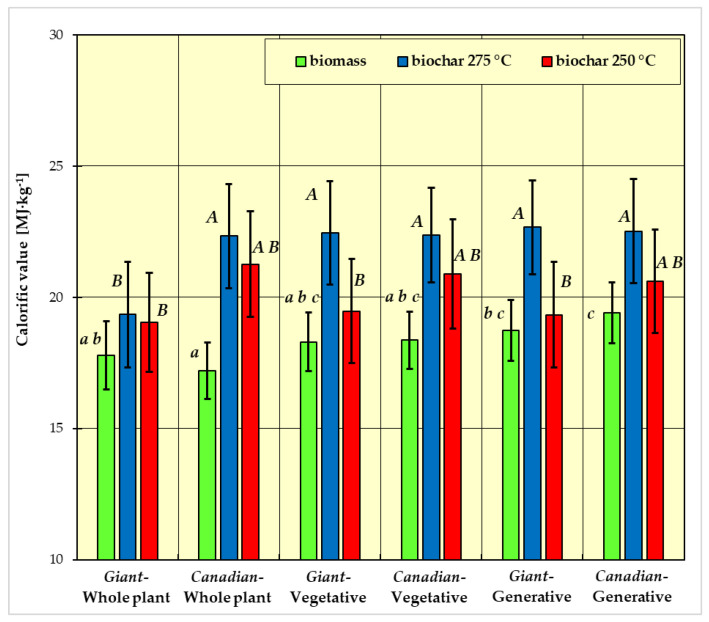
Calorific value of biomass and biochar. All data are expressed as mean ± SD. Bars with a different letter indicate significant differences according to Fisher’s LSD test (*p* < 0.05 was accepted as statistically significant). Homogeneous groups are marked with the same letters (lowercase letters *a*, *b*, *c* for biomass homogeneous groups, uppercase letters *A*, *B* for biochar homogeneous groups).

**Figure 4:**
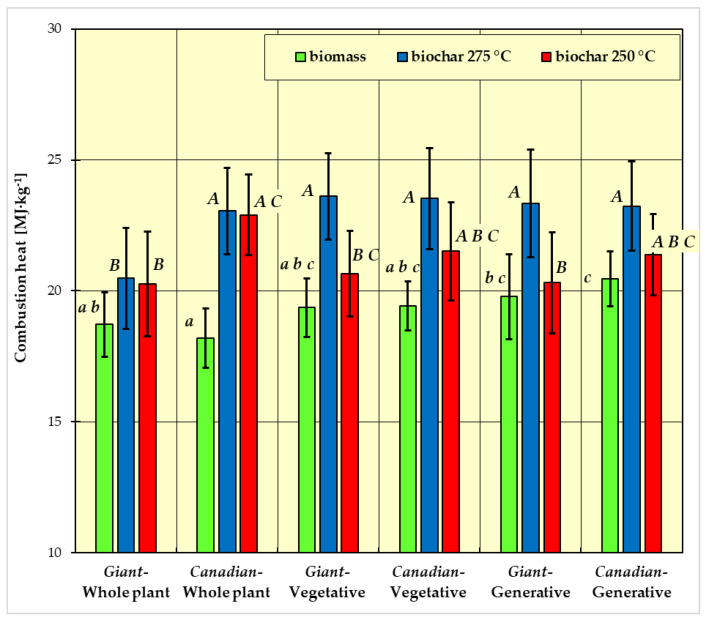
Combustion heat of biomass and biochar. All data are expressed as mean ± SD. Bars with a different letter indicate significant differences according to Fisher’s LSD test (*p* < 0.05 was accepted as statistically significant). Homogeneous groups are marked with the same letters (lowercase letters *a*, *b*, *c* for biomass homogeneous groups, uppercase letters *A*, *B*, *C* for biochar homogeneous groups).

In Figure 1, Figure 2, Figure 3 and Figure 4, the measurement results for biomass and biochar for all dependent variables (ash content, volatile matter content, calorific value, and combustion heat) are presented as mean values from five independent measurements ± standard deviation (SD).

The results of Fisher’s LSD test for the dependent variables are presented in Figure 1, Figure 2, Figure 3 and Figure 4, respectively. Homogeneous groups are marked with the same letters (lowercase letters for biomass homogeneous groups, uppercase letters for biochar homogeneous groups). Statistical analysis was performed for *p* <0.05.

Based on obtained results, it was found that ash content in the biomass (Figure 1) ranged from approx. 5 to 20%. The highest ash content was found in the whole plant of the *Giant* goldenrod, and the lowest in the generative and vegetative parts of both species. Ash content in the generative and vegetative parts was similar for both species at around 5%. Ash content in the *Canadian* goldenrod—whole plant was approx. 10%.

The average content of volatile matter in the tested biomass ranged from about 65 to 80% (Figure 2). The whole plant of the *Giant* goldenrod had the lowest content of volatile matter, and the highest was found in the generative part of the *Canadian* goldenrod. In the *Canadian* goldenrod—whole plant, volatile matter content was about 74%. In the remaining studied material (the vegetative parts of the *Giant* and *Canadian* goldenrod and the generative part of the *Giant* goldenrod), content ranged between 77% and 78%.

After taking all parameters into account, the calorific value of biomass ranged from approx. 17 to approx. 21 MJ∙kg^−1^ (Figure 3). The highest calorific value was found in the generative part of the *Canadian* goldenrod and the lowest in the *Canadian* goldenrod—whole plant. The calorific value of the *Giant* goldenrod—whole plant was approximately 18 MJ∙kg^−1^, and in the vegetative parts of both species and the generative part of the *Giant* goldenrod, it ranged between approximately 18–19 MJ∙kg^−1^.

Figure 4 shows the average heat of biomass combustion obtained during the measurements. The highest value of the heat of combustion was characteristic for the generative part of the *Canadian* goldenrod (approx. 20.5 MJ∙kg^−1^), and the lowest for the whole plant of the *Canadian* goldenrod (approx. 17 MJ∙kg^−1^). The heat of combustion of the whole plant of the *Giant* goldenrod was equal to approximately 18 MJ∙kg^−1^, and approximately 19 MJ∙kg^−1^ for the generative and vegetative part of the *Giant* goldenrod as well as the vegetative part of the *Canadian* goldenrod.

The ash content in biochar obtained after biomass torrefaction increased significantly. No major differences were observed in the ash content in the biochar obtained at 250 °C and 275 °C (Figure 1). In the first case (250 °C), the highest values were recorded for biochar obtained from whole plants (24% for the *Giant* goldenrod and about 20% for the *Canadian* goldenrod). The lowest value ranged from slightly more than 7% to less than 11% and was higher for the vegetative part of both cultivars. On the other hand, for the higher temperature (275 °C), the average ash content ranged from 7% to over 24% and was also higher for the vegetative part of the plant of both species.

The volatile matter content decreased after the roasting process (Figure 2). By analyzing the volatile matter content of the biochar produced at a temperature of 250 °C, it can be seen that a smaller amount of volatile matter occurs in the vegetative part of *Giant* goldenrod compared to *Canadian* goldenrod; about 41% and 52%, respectively (Figure 2). Volatile matter content in biochar obtained at 275 °C ranged from 40% to 54%. The highest volatile matter content was found in the vegetative part of *Canadian* goldenrod, and the lowest in the whole plant of both species.

As a result of torrefaction, the calorific value and heat of combustion increased. Of the samples obtained at a torrefaction temperature of 250 °C, the lowest calorific value was found for *Giant* goldenrod in the generative part (19.34 MJ∙kg^−1^), while the highest was for *Canadian* goldenrod in the vegetative part (20.86 MJ∙kg^−1^) (Figure 3). The average calorific value of torrefaction obtained at the temperature of 275 °C ranged from 15.47 to 22.68 MJ∙kg^−1^. The lowest value was 15.47 MJ∙kg^−1^ for the whole plant of *Giant* goldenrod, while the highest value was 22.68 MJ∙kg^−1^ for *Canadian* goldenrod in the generative part.

The heat of combustion of biochar formed at 250 °C ranged from 20.26 to 22.90 MJ∙kg^−1^ (Figure 4). The lowest heat of combustion was found for the whole plant of *Giant* goldenrod, with the highest value for the vegetative part of *Giant* goldenrod. The average individual values of the heat of combustion for biochar obtained at 250 °C and 275 °C are shown in Figure 4.

The ANOVA results for biomass (Table 3) show a statistically significant effect of the independent variable plant on all four dependent variables, while the independent variable species has a statistically significant effect on the dependent variables ash content and volatile matter content. For the ash content and volatile matter content variables, there is a statistically significant interaction of the species * plant factors.

According to the results of Fisher’s post-hoc LSD test for ash content (Figure 1), there were three homogeneous groups for biomass, which differ statistically significantly from one another (*p* < 0.05):

- group a: *Giant* goldenrod—plant vegetative, *Canadian* goldenrod—plant vegetative, *Giant* goldenrod—plant generative, and *Canadian* goldenrod—plant generative;
- group b: *Canadian* goldenrod—whole plant;
- group c: *Giant* goldenrod—whole plant.

For the dependent variable volatile matter content, three homogeneous groups were also distinguished for biomass (Figure 2):

- group a: *Giant* goldenrod—plant vegetative, *Canadian* goldenrod—plant vegetative, *Giant* goldenrod—plant generative and *Canadian* goldenrod—plant generative;
- group b: *Canadian* goldenrod—plant vegetative and *Canadian* goldenrod—whole plant;
- group c: *Giant* goldenrod—whole plant.

In the case of the dependent variables calorific value (Figure 3) and combustion heat (Figure 4), there is no such clear differentiation for biomass. There are also three homogeneous groups:

- group a: *Giant* goldenrod—whole plant, *Canadian* goldenrod—whole plant, goldenrod *Giant*-plant vegetative and goldenrod *Canadian*-plant vegetative;
- group b: goldenrod *Giant*-whole plant, goldenrod *Giant*-plant vegetative, *Canadian* goldenrod—plant vegetative and *Giant* goldenrod—plant generative;
- group c: *Giant* goldenrod—plant vegetative, *Canadian* goldenrod—plant vegetative, *Giant* goldenrod—plant generative and *Canadian* goldenrod—plant generative.

The ANOVA results for biochar (Table 4) show a statistically significant effect of the independent variable species on all four dependent variables. The independent variable plant has a statistically significant effect on the dependent variables’ ash content and volatile matter content. The independent variable material has a statistically significant effect on the dependent variables volatile matter content, calorific value, and heat of combustion. For the ash content and volatile matter content variables, there is a statistically significant interaction of the species*plant factors.

The results of the post-hoc Fisher’s LSD test conducted for biochar indicated that for ash content (Figure 1), five homogeneous groups differ statistically significantly from each other (*p* <0.05):

- group A: *Canadian*-generative-250 °C, *Canadian*-generative-275 °C, *Giant*-generative-250 °C, *Giant*-generative-275 °C;
- group B: *Canadian*-vegetative-250 °C, *Canadian*-vegetative-275 °C, *Giant*-vegetative-250 °C, *Giant*-vegetative-275 °C;
- group C: *Giant*-generative-275 °C, *Canadian*-vegetative-275 °C;
- group D: *Canadian*-whole plant-250 °C, *Canadian*-whole plant-275 °C;
- group E: *Giant*-whole plant-250 °C, *Giant*-whole plant-275 °C.

For the dependent variable volatile matter content, seven homogeneous groups were distinguished for biomass (Figure 2):

- group A: *Canadian*-generative-250 °C, *Canadian*-generative-275 °C, *Giant*-generative-250 °C, *Giant*-generative-275 °C;
- group B: *Giant*-whole plant-250 °C, *Giant*-whole plant-275 °C, *Canadian*-whole plant-250 °C;
- group C: *Giant*-whole plant-250 °C, *Giant*-whole plant-275 °C, *Canadian*-whole plant-275 °C;
- group D: *Giant*-whole plant-275 °C, *Canadian*-whole plant-275 °C, *Giant*-vegetative-250 °C;
- group E: *Giant*-vegetative-275 °C;
- group F: *Canadian*-vegetative-250 °C;
- group G: *Canadian*-vegetative-275 °C.

For biochar, there were three homogeneous groups in the dependent variables calorific value (Figure 3) and combustion heat (Figure 4):

- group A: *Canadian*-generative-250 °C, *Canadian*-generative-275 °C, *Giant*-generative-275 °C, *Canadian*-vegetative-250 °C, *Canadian*-vegetative-275 °C, *Giant*-vegetative-275 °C, *Canadian*-whole plant-250 °C, *Canadian*-whole plant-275 °C;
- group B: *Canadian*-generative-250 °C, *Giant*-generative-250 °C, *Canadian*-vegetative-250°, *Giant*-vegetative-250 °C, *Giant*-whole plant-250 °C, *Giant*-whole plant-275 °C;
- group C: *Canadian*-generative-250 °C, *Canadian*-vegetative-250 °C, *Giant*-vegetative-250 °C, *Canadian*-whole plant-250 °C.

## 4. Discussion

The moisture content of individual studied species and their parts did not differ significantly. Similar moisture content was found for grassy plants, herbal plants, and weeds (miscanthus 11.5%, millet—13 to 15%) [13]. After the torrefaction process, the biochar moisture content decreased as a result of water evaporation during thermal biomass treatment. The differences in the moisture content in fresh biomass and biochar of both species had an impact mainly on the calorific value. The level of average moisture content in individual parts of plants remained at a similar level.

The species of tested plants and the parts of these plants had a significant impact on the ash content. The ash content is the sum of the residues from the thermal decomposition of organic materials and non-flammable minerals as a result of an organic matter reduction process that breaks down matter and degasses it. The ash content in individual parts of the plants varies due to the different chemical compositions and structures of individual plants. The highest ash content was found in the whole plant for each of the tested species and the values of ash content differed significantly between species. As a result of torrefaction, the ash content increased to 24% in biochar from the *Giant* goldenrod and 20% from the *Canadian* goldenrod. There was a statistically significant difference in the ash content for both species of goldenrod between the whole plants, their vegetative part, and their generative part. On the other hand, the ash content did not differ significantly between the species in their respective plant parts. Two homogeneous groups were formed—one consisting of vegetative and the other, generative parts.

The increase in ash content was also observed in the torrefaction process of other biomass species [45]. McKendry [13] reported that the ash content of the miscanthus is 2.8% and switchgrass is 4.5%. Chen et al. [46] investigated the ash content in fresh biomass (coffee waste, sawdust, and rice husks) and biochar obtained from torrefaction at temperatures of 240 °C and 270 °C. The ash content in these materials was much lower compared to the goldenrod, but the direction of changes after torrefaction was similar—the ash content in biochar increased. The high ash content of both goldenrod species was due to soil contamination of the plants. Due to the acceptable ash content level, biochar from both types of goldenrod can be used in energy and agriculture.

As a result of torrefaction, the content of volatile substances in biochar decreased. Fresh biomass contained about 80% volatile matter, and after torrefaction, decreased to about 40%, regardless of the plant variety, part, or the process temperature. Such changes in the content of volatile matter in the process of biomass torrefaction were observed for other plants, and a similar trend of changes was observed in the case of biochar obtained from other types of biomasses, such as miscanthus, wheat straw, or switchgrass [13,47]. Chen et al. observed a tendency for such changes in the content of volatile matter as a result of torrefaction of cotton stalks [48]; the content of volatile matter in the cotton stalk decreased from approx. 75% to approx. 56%. Similar to the ash content, the content of volatile matter significantly depended on the plant part and species (Table 3). In the case of biochar, a similar relationship was observed. The content of volatile substances is an important parameter that significantly affects the combustion process [49]. Biomass contains up to 2.5 times more volatile matter than coal, which affects the conditions of its ignition and combustion [50]. Combustion of fuel with a high volatile content (containing a large number of combustible substances) creates a large flame and more air should be introduced for the fuel to burn completely.

As in the case of the volatile matter and ash content, the calorific value and heat of combustion of fresh biomass are primarily influenced by whole plants of goldenrod of both studied species. It was observed that the calorific value and heat of combustion for the tested samples of *Canadian* goldenrod were higher compared to the values obtained for *Giant* goldenrod. When analyzing the measurement results of all fresh biomass samples, the highest calorific value and heat of combustion were found for the vegetative part of *Canadian* goldenrod, and the lowest for the sample from the whole plant of *Giant* goldenrod. This most likely results from a different chemical structure (the content of cellulose, hemicellulose, and lignin) and, above all, from the share of elements such as carbon and hydrogen, which have a significant impact on these studied values and material moisture content [47].

It was also observed that the biochar produced at a temperature of 275 °C had higher values of heat of combustion compared to the second variant of the experiment. The reason for this could be an increase in the carbon content of the material. For the remaining biomass species, an increase in calorific value and heat of combustion was observed [13,45]. Compared to fossil coal, the calorific value of the biochar obtained from goldenrod is similar.

The ash content in the individual parts of plants varied, with the highest found in samples from whole plants (Figure 1). Differences depending on the species were also observed (for biomass from *Giant* goldenrod—approx. 20%, and for biomass from *Canadian* goldenrod—10%). As a result of torrefaction of whole plant samples, the ash content increased to 24% for *Giant* goldenrod biochar and 20% for *Canadian* goldenrod biochar.

The increase in ash content was also observed in the process of torrefaction of other species of biomass [45]. McKendry [13] reported that the ash content in miscanthus is 2.8%, and in switchgrass—4.5%.

As a result of torrefaction, the content of volatile matter decreased. Fresh biomass contained about 80% of volatile matter, and after torrefaction, this decreased to about 40%, regardless of the variety or part of the plant and the temperature of the process. Such tendencies for changes in the content of volatile matter in the process of biomass torrefaction were observed for other plants, and the results of research on the torrefaction process of miscanthus and switchgrass by McKendry [13] may serve as an example of this. Chen et al. observed such a tendency in the changes to volatile matter content as a result of torrefaction of cotton stalks [48]. The volatile matter content in the cotton stalk decreased from approx. 75% to approx. 56%.

The calorific value of biochar depends on the temperature of the torrefaction process—it is higher for a higher process temperature (Figure 3). For torrefaction carried out at 275C, the lowest calorific value was obtained for the whole plant of *Giant* goldenrod, while for the other variants of the experiment, the calorific value was significantly higher and similar in value. For the torrefaction carried out at 250 °C, greater differentiation of the calorific value was observed for different samples. This is probably due to incomplete degradation of compounds such as cellulose, hemicellulose, and lignin. The lowest level of calorific value was obtained for *Giant* goldenrod in generative parts and the highest for *Canadian* goldenrod in vegetative parts.

Comparing the results for the calorific values of the obtained biochar (19–23 MJ∙kg^−1^) with the calorific values for wood, it can be noted that they are higher or at least comparable. The calorific value of wood ranges from 17.4 to 18.5 MJ∙kg^−1^ [51,52,53]. Compared with solid fossil fuels, the calorific value of the obtained torrefaction from goldenrod is similar. The calorific value of hard coal is 20.9–33.4 MJ∙kg^−1^ [54,55,56,57] and brown coal (lignite) 5.9–24.9 MJ∙kg^−1^ [58,59,60,61,62].

For the heat of combustion measured in our research, the trends of changes are similar to the calorific value (Figure 4).

The regularities obtained by us regarding the calorific value and heat of combustion are confirmed by the results of studies of other types of biomasses [13,45].

The measured bulk density of biomass and biochar was different for individual parts of the plant as well as for individual species. After the torrefaction process, this density decreased for each part of the plant tested.

Goldenrod is lignocellulosic biomass, a natural bio-composite formed by cellulose, hemicellulose, and lignin. During roasting, the torrefaction process transforms the chemical and structural features of the biomass, which affect the quality of the final product—biochar. Lignin decomposition begins at temperatures above 200 °C, with slower kinetics than that of hemicellulose or cellulose [63].

Hemicellulose undergoes a thermochemical transformation in the temperature range from 225–325 °C, and cellulose at temperatures of between 325–375 °C. During torrefaction, hemicellulose and, to some extent, lignin are broken down, depending on their chemical structure (cellulose, hemicellulose, and lignin content) and type of biomass [10]. Research is being conducted to improve the torrefaction process of various types of biomasses (bamboo, agricultural waste—coffee residues, sawdust, rice husks) by understanding the mechanisms of chemical and structural changes that have a significant impact on energy efficiency, mass, and physical and chemical properties [46,64,65]. Therefore, in the case of the presented research, it is necessary to extend the scope of measurements to include studying the chemical composition and molecular structure of the material subjected to torrefaction at different temperatures. Such research will make it possible to explain the differences in the physical and energy properties observed between the vegetative and generative parts of two goldenrod species and the whole plant.

## 5. Conclusions

Based on the results, it was found that the whole plant of both goldenrod species had a significant impact on the ash content and volatile matter content in fresh biomass and biochar. The ash content in fresh biomass was the highest in the generative part of the *Giant* goldenrod and the lowest in the generative part of the *Canadian* goldenrod.

After biomass torrefaction, the ash content increased. It did not, however, differ significantly for individual biochars obtained from samples calcined at two different temperatures. The highest ash content in biochar was obtained from whole plant samples of both species, the lowest in biochar from the generative part of *Canadian* goldenrod and *Giant* goldenrod. The volatile matter content in goldenrod was the highest in the generative part of the *Canadian* goldenrod and the lowest in the whole plant of the *Giant* goldenrod. After torrefaction, the volatile matter content decreased—the lowest volatile matter content was found in the whole plants of both species and the highest in the vegetative part of the *Canadian* goldenrod.

The calorific value and heat of combustion of biomass and biochar are highly dependent on the type of sample (whole plant, generative part, vegetative part). For biochar produced at a higher temperature, the calorific value and heat of combustion are both higher.

## Figures and Tables

**Table 1:** Biomass and biochar bulk density.

Species/Plant Part	Biomass Bulk Density [kg∙m^−3^]	Biochar Bulk Density [kg∙m^−3^]
*Giant* goldenrod/whole plant	464–482	229–301
*Giant* goldenrod/vegetative part	395–405	149–291
*Giant* goldenrod/generative part	361–452	188–253
*Canadian* goldenrod/whole plant	470–487	243–324
*Canadian* goldenrod/vegetative part	331–376	178–245
*Canadian* goldenrod/generative part	357–465	193–287

**Table 2:** Biomass and biochar moisture content.

Species/Plant Part	Biomass Moisture Content [%]	Biochar 275 °C Moisture Content [%]	Biochar 250 °C Moisture Content [%]
*Giant* goldenrod /whole plant	9.18–10.73	5.19–6.08	5.28–6.19
*Giant* goldenrod /vegetative part	11.9–12.86	7.31–7.99	7.24–7.94
*Giant* goldenrod /generative part	11.01–12.20	8.34–9.37	8.09–8.96
*Canadian* goldenrod /whole plant	10.76–12.22	6.53–6.98	6.41–7.05
*Canadian* goldenrod /vegetative part	11.30–12.07	7.14–8.07	7.45–8.24
*Canadian* goldenrod /generative part	11.70–12.57	7.26–8.03	7.35–7.99

**Table 3:** ANOVA results for ash content, volatile matter content, calorific value, and combustion heat in samples of *Canadian* and *Giant* goldenrod biomass.

Intergroup Factors	*p* Ash Content [%]		*p* Volatile Matter Content [%]		*p* Calorific Value [MJ∙kg^−1^]		*p* Combustion Heat [MJ∙kg^−1^]	
Species	0.000008	*	0.000550	*	0.925095		0.868240	
Plant part	0.000000	*	0.000000	*	0.019775	*	0.017319	*
Species * Plant part	0.000000	*	0.000005	*	0.484488		0.542392	

Significant results are indicated by asterisks * (*p* < 0.05).

**Table 4:** ANOVA results for ash content, volatile matter content, calorific value, and combustion heat in samples of *Canadian* and *Giant* goldenrod biochar.

Intergroup Factors	*p* Ash Content [%]		*p* Volatile Matter Content [%]		*p* Calorific Value [MJ∙kg^−1^]		*p* Combustion Heat [MJ∙kg^−1^]	
Species	0.001345	*	0.000000	*	0.015063	*	0.016005	*
Plant part	0.000000	*	0.000000	*	0.344569		0.513122	
Material	0.795809		0.000771	*	0.000640	*	0.000596	*
Species *Plant part	0.001258	*	0.000000	*	0.187318		0.098905	
Species *Material	0.825913		0.299548		0.488718		0.435784	
Plant part *Material	0.844045		0.137445		0.263790		0.078582	
Species *Plant part *Material	0.994026		0.582448		0.588975		0.876665	

Significant results are indicated by asterisks * (*p* < 0.05).

## Data Availability

Not applicable.
